# Spaceflight multi-omics reveals vulnerabilities of human germ cell development

**DOI:** 10.1126/sciadv.aee0143

**Published:** 2026-07-15

**Authors:** Ying Li, Hui Gao, Jie Xiong, Nan Wang, Yongchun Yuan, Linjun Wang, Jinzhong Xu, Wenze Huang, Gikin Tan, Xiaojuan He, Qiangfeng Cliff Zhang, Tao Zhang, Kehkooi Kee

**Affiliations:** ^1^ The State Key Laboratory for Complex Severe and Rare Diseases; Beijing Key Laboratory of Intelligent Organ Biofabrication and Regenerative Repair; SXMU-Tsinghua Collaborative Innovation Center for Frontier Medicine; School of Basic Medical Sciences, Tsinghua Medicine, Tsinghua University, Beijing, 100084, China.; ^2^ School of Medicine, Fuzhou University, Fuzhou 350108, China.; ^3^ Shanghai Institute of Technical Physics of the Chinese Academy of Sciences, Shanghai 200083, China.; ^4^ Key Laboratory of Space Utilization, Technology and Engineering Center for Space Utilization, Chinese Academy of Sciences, Beijing 100094, P.R. China.; ^5^ MOE Key Laboratory of Bioinformatics, Center for Synthetic and Systems Biology, School of Life Sciences, Tsinghua University, Beijing, China.

## Abstract

Understanding the impact of spaceflight on human reproduction is critical for interplanetary exploration, yet technical barriers have limited direct studies of germ cell biology in orbit. Here, we utilized an automated bioreactor that supported long-term differentiation of human embryonic stem cells into human induced primordial germ cells (hiPGCs), human induced ovarian follicles (hiOFs), and human induced spermatogonial stem cells (hiSSCs) aboard spacecraft. Integrated real-time imaging, programmable medium perfusion, and in situ preservation enabled time-resolved multi-omics analysis. During missions on China’s Tianzhou-1 and Tianzhou-6 spacecraft, spaceflight reduced hiPGC specification efficiency by approximately 50% and suppressed hiSSC proliferation by 26%. Transcriptome-translatome coordination revealed cell-type-specific dysregulation of extracellular matrix organization, microtubule dynamics, and lipid metabolism. Whole-exome sequencing and DNA methylome analysis demonstrated preserved genomic integrity despite these functional perturbations. These findings provide direct evidence that spaceflight perturbs human germ cell development and establish a scalable framework for monitoring cellular adaptation during deep-space missions.

## Teaser

Spaceflight impairs human germ cell development in orbit, revealing reproductive vulnerabilities for deep-space exploration.

## INTRODUCTION

Humanity has entered a new era of space exploration, with increasing attention focused on the impact of the spaceflight environment on astronaut health (*1*–*5*). Space radiation and microgravity loom as principal threats during extended missions, contributing to a spectrum of physiological challenges (*3*, *6*). In addition to the established carcinogenic risk associated with prolonged radiation exposure (*7*), studies have reported bone demineralization and muscle atrophy (*8*, *9*), central nervous system alterations (*10*, *11*), immune system dysfunction (*5*, *6*, *12*–*14*), and adverse cardiovascular outcomes (*15*–*19*). However, despite these systemic physiological studies, the impact of spaceflight on human germ cells remains largely unexplored (*20*).

Germ cells, which transmit genetic information across generations (*21*, *22*), are fundamental to human reproduction and critical for sustaining the species during long-term space habitation. Prenatal germ cell development establishes the epigenetic and structural foundation for adult reproductive potential. Proper development of the germline is essential for maintaining genomic integrity under the combined stresses of cosmic radiation, microgravity, and circadian disruption. Current knowledge of spaceflight-associated reproductive effects derives largely from studies of endocrine parameters in astronauts (*23*, *24*) or animal models (*25*, *26*). For instance, studies have shown that microgravity disrupts estrogen cycles in female rodents (*27*), impair sperm production in male mice (*28*), and suppress systemic testosterone levels in male rats (*26*, *29*). However, the direct impact of the space environment on the developmental trajectory of human germ cells remains poorly defined, constrained by both ethical considerations and limited sample availability.

In vitro differentiated cells provide a powerful model for exploring developmental responses to spaceflight (*20*), but the induction of human germ cells under spaceflight conditions has not been achieved. To address this, we applied established protocols for the stage-specific differentiation of human germ cells to generate three distinct germ-like lineages from human embryonic stem cells (hESCs) under spaceflight conditions: hiPGCs (*30*), hiOFs (*31*), and hiSSCs (*32*). Together, these models capture key developmental stages: hiPGCs represent early development of human bipotential germ cells, hiOFs model female germline development, and hiSSCs reflect the initial phase of male germ cell differentiation.

Multi-omics sequencing technologies are indispensable for capturing the molecular landscape of germ cells, in which post-transcriptional regulation plays a central role in gametogenesis (*33*–*36*). In oocytes, transcriptional silencing precedes maturation, with the extensive translation of pre-stored mRNAs occurring only after meiosis resumption (*35*, *37*, *38*). During spermiogenesis, liquid-liquid phase separation (LLPS)-mediated post-transcriptional regulation activates translationally inert mRNPs by organizing target mRNAs into FXR1-containing granules (*34*). Translatome analysis thus emerges as a critical tool for resolving the precise regulatory dynamics of germline development (*33*, *34*, *39*–*41*). Furthermore, spaceflight environmental stressors, including microgravity and radiation, can perturb post-transcriptional networks and induce cumulative epigenetic and genomic alterations (*42*). Studies have shown that radiation increases mutation rates in *Caenorhabditis elegans* (*43*) and mammals (*44*–*47*), whereas researchers have documented DNA methylation changes in *Mus musculus* (*1*) and human T-cells (*3*) exposed to spaceflight conditions. Therefore, a combined analysis of the DNA methylome, whole-genome, transcriptome, and translatome within the same cell population is essential for a comprehensive understanding of the spaceflight effects.

In this study, we combined an automated cell-culture and imaging platform with live-cell imaging and multi-omics profiling to examine human germ cell development under spaceflight conditions. We identified a spaceflight-induced vulnerability characterized by cell type-specific disruption of extracellular matrix organization and translational reprogramming, while genomic integrity remains largely preserved. Together, these findings provide evidence that spaceflight perturbs human germline development and establish a framework for future mechanistic investigation.

## RESULTS

### Construction of an automated system for human embryonic stem cell differentiation, imaging, and sample collection

**Fig. 1.**
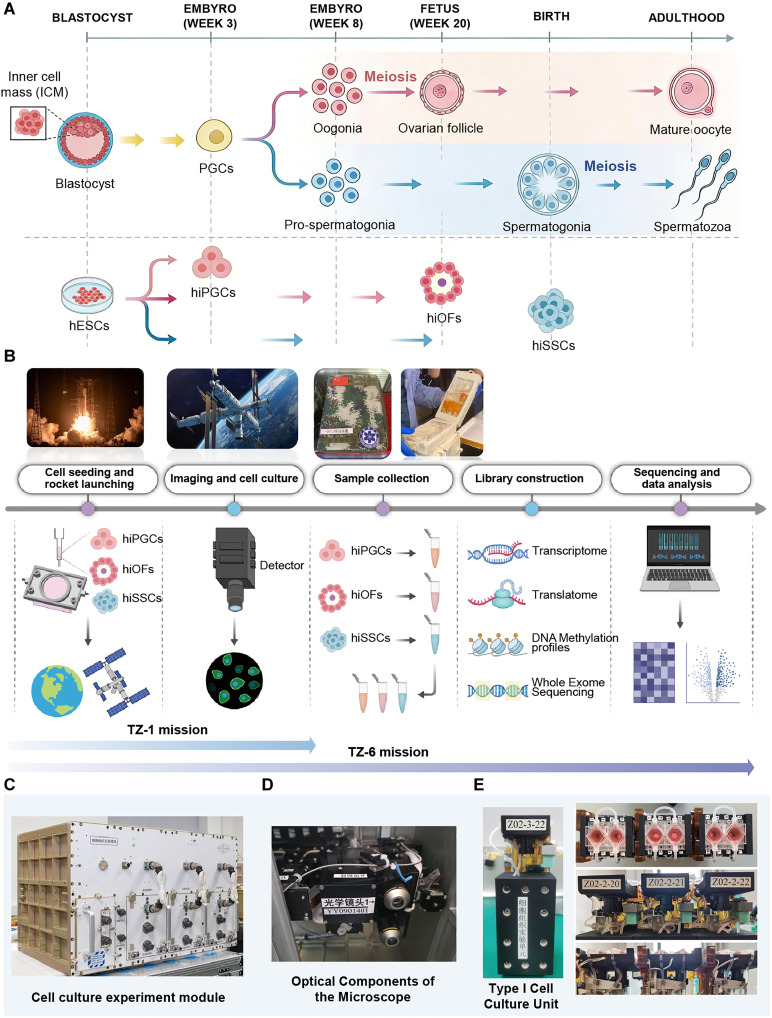
Experimental platform and workflow for automated culture of hESC-derived germ cell lineages for spaceflight experiment. (**A**) Schematic of human germ cell development and the corresponding hESC-derived lineage differentiation trajectory. (**B**) Schematic of the experimental design and workflow for the TZ-1 and TZ-6 missions. (**C**) Automated cell culture system-integrated experimental cabinet. (**D**) Optical microscopy module of the experimental cabinet. (**E**) Type I cell culture unit. Left, overall view of the culture unit; right, top-to-bottom views of the mounted culture chamber and the peristaltic pump with tubing connections (green box, pump mounting position).

To investigate the effects of spaceflight on the development of germ cells derived from hESCs, we constructed an automated cell culture bioreactor with real-time imaging capabilities. This system enabled the cultivation of hiPGCs, hiOFs, and hiSSCs under spaceflight conditions, as well as the preservation and recovery of samples for post-flight analysis. The hiPGCs, hiOFs, and hiSSCs correspond to embryonic germ cells prior to sexual differentiation, female ovarian follicle, and male spermatogonial stem cells, respectively (Fig. 1A). Upon the samples returned to Earth, we analyzed the spaceflight-differentiated cells using transcriptome, translatome, DNA methylation, and whole-exome sequencing (Fig. 1B). During the Tianzhou-1 (TZ-1) mission (launched 20 April 2017, 380 km orbital altitude), we achieved a 30-day in-orbit differentiation culture of hiPGCs induced from hESCs. Live imaging confirmed viability and germ-cell identity, but the non-recoverable setup precluded molecular characterization (*48*–*50*). In contrast, the Tianzhou-6 (TZ-6) mission (launched 10 May 2023) resolved these limitations and facilitated the differentiation of hiPGCs, hiOFs, and hiSSCs under spaceflight conditions, enabling fluorescence imaging with real-time data transmission and the acquisition of matched multi-omics datasets from both spaceflight and ground-control groups.

To support automated cell culture and sample preservation in spaceflight environment, we designed the orbital bioreactor system comprising two functionally integrated modules (fig. S1A, Fig. 1C). The cell culture support module maintained a 5% CO_2_ concentration through gas-permeable membrane regulation, while the culture execution module incorporated phase-contrast microscopy (fig. S1B, Fig. 1D), dual-wavelength fluorescence imaging (488/561 nm excitation), and temperature-regulated culture chambers maintaining 36 ± 1°C. To accommodate distinct differentiation timelines, we engineered specialized culture units: Type I configurations supporting 6-day hiPGC/hiOF induction and Type II units enabling 15-day hiSSC maturation. To ensure microbial containment, we assembled and connected the entire system in a biosafety cabinet (fig. S1C). Each unit integrated thermoelectric heating/cooling plates (±0.5°C precision), peristaltic pump-mediated medium exchange systems, and dual-temperature storage compartments preserving reagents at 10°C and RNA protect solutions at 4°C. Within these units, we cultured cells in the upper chamber, where integrated peristaltic pumps automated medium perfusion (fig. S1D, Fig. 1E).

During the TZ-6 mission, astronauts integrated the automated cell culture device into the Wentian module of the Chinese Space Station, initiating automatic differentiation via programmed pump sequences and capturing time-lapse images at 24-hour intervals for morphological analysis. Prior to the Shenzhou-15 crew return on 4 June 2023, biological samples underwent RNALater fixation at 4°C for 48 hours followed by transfer to −80°C cryogenic storage modules. Ground-control experiments replicated protocols using duplicate systems under Earth-normal gravity, establishing matched parameters for rigorous comparison of spaceflight effects. This experimental framework represents the successful integration of real-time imaging with multi-omics profiling for human germ cell differentiation studies in orbital microgravity environments.

### Comparison of hESC-derived lineage differentiation under spaceflight and ground-control conditions

To systematically investigate the effects of spaceflight on in vitro germ cell development from hESCs, we first examined PGC differentiation during the TZ-1 mission using a reporter system in which the germ cell-specific VASA promoter drove GFP expression (VASA-GFP) (*30*, *31*). This system enabled real-time tracking of PGC differentiation through continuous bright-field and fluorescence live imaging over 30 days, capturing dynamic changes in hiPGC behavior under microgravity (fig. S2A). The automated culture platform supported uninterrupted cell culture throughout the 30-day mission by integrating closed-loop environmental control with live-imaging capabilities, thereby enabling real-time fluorescence monitoring and transmission of experimental data to ground stations. This autonomous system maintained cell viability throughout the month-long experiment while recording dynamic differentiation processes. However, the limited resolution constrained the detection of statistically significant differences in hiPGC differentiation metrics (fig. S2B).

**Fig. 2.**
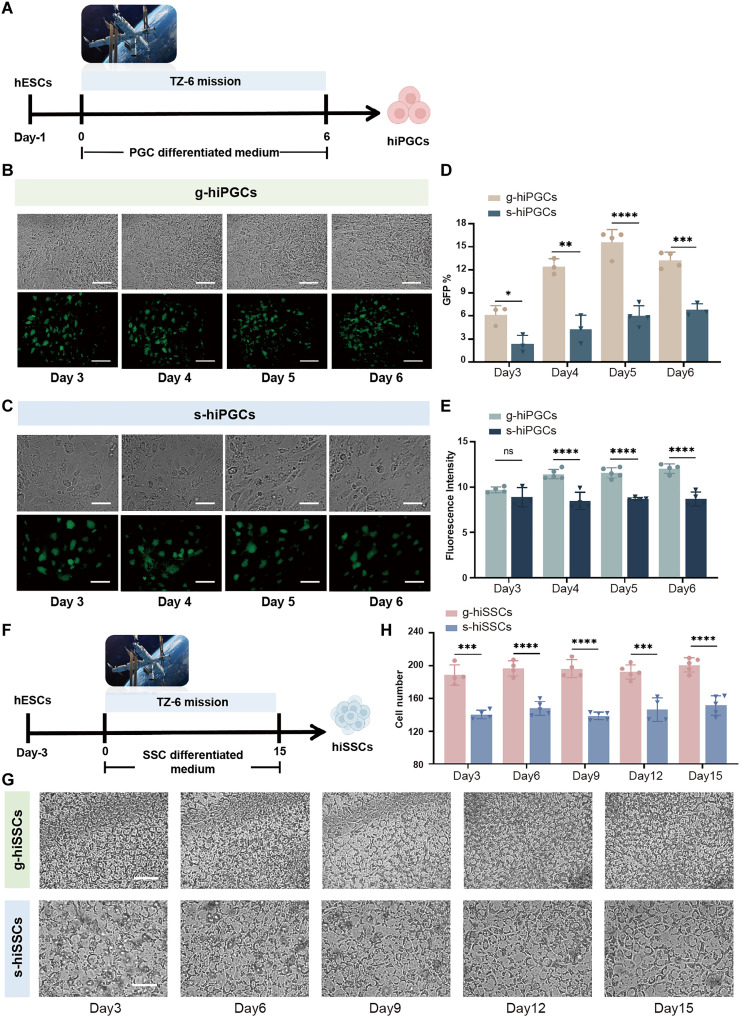
Effect of spaceflight condition on hiPGC morphology and the expression of PGC markers. (**A**) Schematic of the 6-day hiPGC differentiation protocol during the TZ-6 space mission. (**B** and **C**) Representative fluorescence images of cell morphology and VASA-GFP expression during hiPGC differentiation under ground-control (B) and spaceflight (C) conditions. Scale bars, 50 μm. (**D**) Quantification of VASA-GFP+ cell populations, showing divergence in germline commitment efficiency between ground and spaceflight groups. (**E**) Comparison of relative mean fluorescence intensity between ground and spaceflight groups. (**F**) Schematic of the 15-day hiSSC differentiation protocol during the TZ-6 mission. (**G**) Representative phase-contrast images of hiSSC morphogenesis in automated bioreactors under ground-control (top) and spaceflight (bottom) conditions. Scale bars, 50 μm. (**H**) Comparison of cell numbers during hiSSC induction under ground-control and spaceflight conditions. Data are mean ± SD. Two independent biological replicates were included for each condition because of payload constraints during the spaceflight mission. For imaging-based quantification in Fig. 2, D, E, and H, multiple image fields were collected from each biological replicate; *N* indicates the number of image fields analyzed. Statistical significance was determined by Student’s t-test. ns, not significant; *P < 0.05, **P < 0.01, ***P < 0.001, and ****P < 0.0001.

Building on the TZ-1 platform, we further optimized the device during the TZ-6 mission to improve live-imaging quality and include additional cell types and biological replicates. Under ground-control and spaceflight conditions, we differentiated hESCs (XX or XY cell lines) into three germ cell lineages: hiPGCs (Fig. 2A), hiOFs (fig. S2C), and hiSSCs (Fig. 2F).

For ground controls, hiPGCs exhibited a VASA-GFP positivity rate of 6 to 15% on differentiation days 3 to 6, with a peak at day 5 (Fig. 2, B and D), with stable fluorescence intensity at 9–12 (Fig. 2, B and E). In the spaceflight environment, the hiPGC positivity rate reduced to 2–6% (*P* < 0.0001 vs. 6–15% in ground controls), and increased gradually from days 3 to 6 (Fig. 2, C and D), whereas fluorescence intensity remained near 8 (Fig. 2, C and E). Both measures decreased substantially relative to ground controls, indicating impaired PGC specification during spaceflight. In contrast, hiOFs cultured under spaceflight conditions displayed normal morphology and remained viable, with no discernible differences from ground-based controls (fig. S2, D and E). During hiSSC differentiation, ground-based cultures maintained a stable hiSSC population from days 3 to 15, whereas spaceflight-exposed cultures exhibited reduced hiSSC numbers, amounting to a 26% reduction relative to the ground group (Fig. 2, F to H). Together, these results demonstrate both the technical feasibility of performing real-time, multi-lineage differentiation experiments under spaceflight conditions and the lineage-specific effects of spaceflight on germ cell development.

### Transcriptome-translatome profiling reveals spaceflight-specific germ cell differentiation signatures

To delineate the molecular landscape of human germ cell development under spaceflight conditions, we performed transcription-translation dual-omics sequencing (T&T-seq) (*37*) on hiPGCs, hiOFs, and hiSSCs from both spaceflight and ground-control conditions. The principal component analysis (PCA) of the transcriptome and translatome profiles revealed that hiPGCs, hiOFs, and hiSSCs clustered distinctly from hESCs under both conditions. Notably, the separation between spaceflight and ground samples was more pronounced at the translatome level, with greater inter-group segregation and high intra-group reproducibility (fig. S3, A and B). Comparative transcriptomics using hESCs as a reference revealed distinct transcriptional profiles across germ cell lineages. hiPGCs shared 1802 upregulated genes under both conditions, including representative marker genes enriched in TGF-β signaling and meiotic processes (fig. S3C). Similarly, hiOFs exhibited 1475 commonly upregulated transcripts characterizing female germline identity, such as those associated with steroid hormone secretion and oocyte maturation (fig. S3D). Furthermore, hiSSCs showed 2116 upregulated genes linked to extracellular matrix organization and sperm motility, reflecting the molecular signatures of male germ cell differentiation (fig. S3E).

**Fig. 3.**
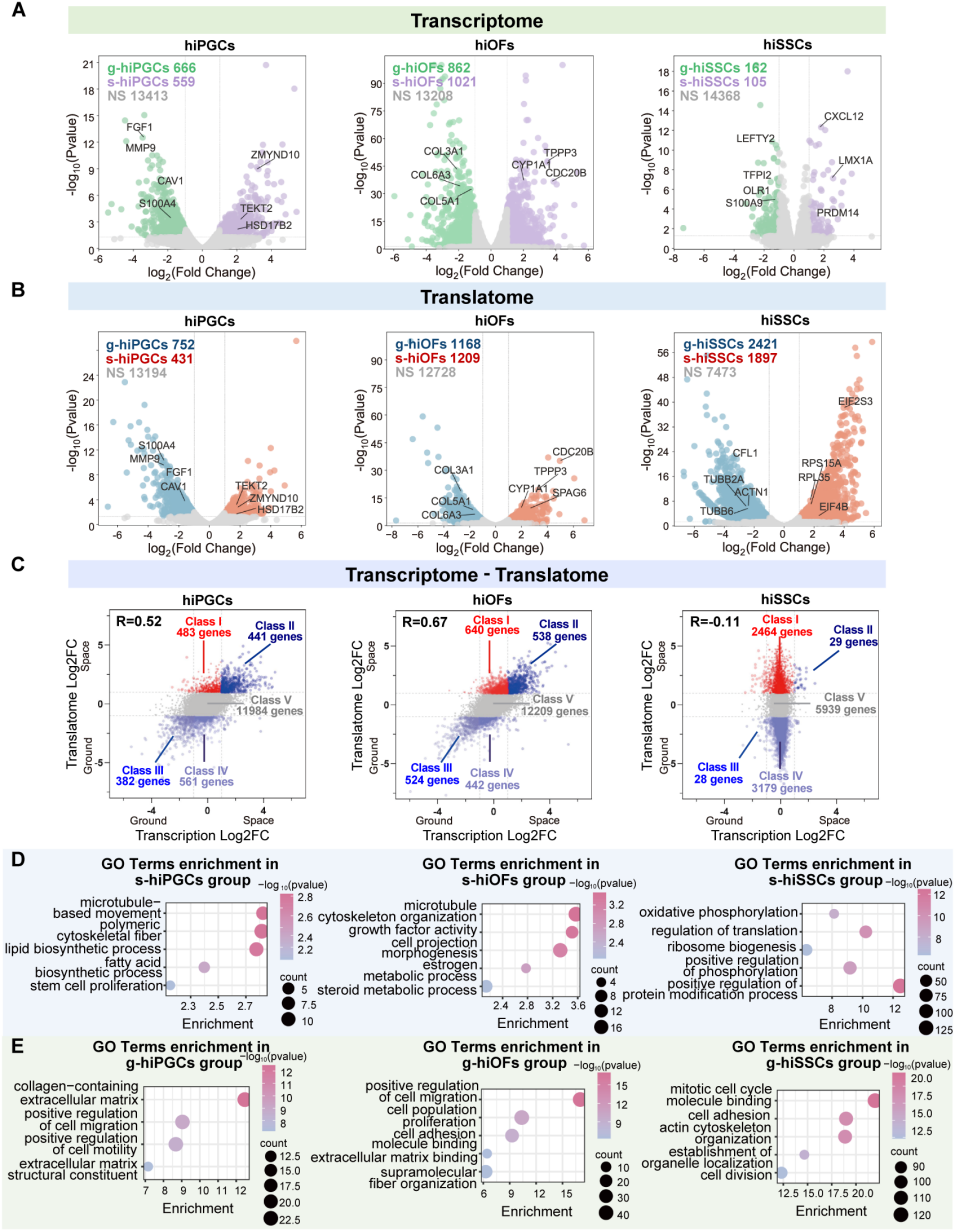
The landscape of potential transcriptome-translatome associations biological processes related to spaceflight. (**A** and **B**) Volcano plots of differentially expressed genes (DEGs) at the transcriptome (A) and translatome (B) levels in hiPGCs (left), hiOFs (middle), and hiSSCs (right) under spaceflight relative to ground control conditions. *P* values were calculated by Wald test. (**C**) Scatter plot of transcriptomic and translatomic changes under spaceflight relative to ground control. Genes were categorized as translationally up-regulated with unchanged transcription (Class I, red), coordinately up-regulated at both transcriptional and translational levels (Class II, blue), coordinately down-regulated at both levels (Class III, bluish gray), or translationally down-regulated with unchanged transcription (Class IV, purple). Pearson correlation coefficients (R) are indicated. (**D** and **E**) GO analysis of significantly enriched gene sets identified from transcriptome-translatome comparisons in hiPGCs (left), hiOFs (middle), and hiSSCs (right) under spaceflight (D) and ground (E) conditions. Enrichment analysis was performed on Class II and Class III genes in hiPGCs and hiOFs, and on Class I and Class IV genes in hiSSCs. Enriched terms are ranked according to -log_10_(*P* value).

Despite retaining core germ cell identity, spaceflight induced lineage-specific transcriptional remodeling. In hiPGCs, 559 transcripts were up-regulated and 666 were down-regulated (*P* < 0.05), with enrichment of microtubule-based movement genes (for example, TEKT2 and ZMYND10) and repression of migration-associated genes (FGF1, CAV1, and MMP9). hiOFs underwent broader transcriptional remodeling, with induction of microtubule cytoskeleton programs (1021 transcripts; TPPP3 and CDC20B) and repression of extracellular matrix genes (862 transcripts; COL3A1, COL5A1, and COL6A3). By contrast, hiSSCs cultured in orbit for 15 days showed comparatively modest transcriptional changes (105 up-regulated and 162 down-regulated transcripts), characterized by activation of morphogenesis-related genes (PRDM14, LMX1A, and CXCL12) and suppression of cell-adhesion genes (OLR1 and S100A9) (Fig. 3A).

To systematically characterize transcriptomic differences between spaceflight and ground-control conditions across the three germ cell lineages, we conducted pathway enrichment analysis using Gene Set Enrichment Analysis (GSEA)/Reactome Comparative analysis revealed distinct molecular signatures: g-hiPGCs exhibited significant enrichment in ECM organization, integrin cell surface interactions and collagen formation pathways, whereas s-hiPGCs demonstrated activation of DNA repair mechanisms such as homologous DNA pairing and double-strand break processing (fig. S4A), suggesting a coordinated DNA damage response and disruption of ECM organization under spaceflight conditions. Microgravity induced similar ECM-related alterations in hiOFs, significantly downregulating collagen chain trimerization and ECM proteoglycan pathways. Notably, spaceflight samples showed marked upregulation of translation-related processes and mitochondrial translation, indicating a translational adaptation to spaceflight stress (fig. S4B). In hiSSCs, spaceflight induced comparable translational alterations, significantly enriching eukaryotic translation initiation and elongation pathways while downregulating genes involved in gap junction assembly (fig. S4C). Interestingly, both hiOFs and hiSSCs demonstrated significant enrichment of translation-associated genes under spaceflight conditions. We therefore examined translatome-wide changes across all three cell types.

Translatome profiling further revealed distinct regulatory responses across the three germ cell lineages. In hiPGCs and hiOFs, translational changes were broadly concordant with transcriptional trends. hiPGCs showed 431 ribosome-associated transcripts with increased abundance and 752 with decreased abundance, whereas hiOFs exhibited 1209 upregulated and 1168 downregulated translated transcripts. In contrast, hiSSCs displayed substantially broader translatome remodeling, with 1897 upregulated transcripts, including translation-related factors such as EIF2S3, EIF4B, and RPL35, and 2421 downregulated transcripts, including mitotic cell cycle-associated genes such as CFL1, TUBB2A, and TUBB6 (Fig. 3B). These findings are consistent with the reduced proliferative capacity of hiSSCs in spaceflight, as reflected by the 26% decrease in cell number (Fig. 2H).

Integrated transcriptomic and translatomic analyses revealed cell type-specific dysregulation. Notably, hiPGCs and hiOFs exhibited coordinated disruption of ECM- and microtubule-related programs at both the transcriptomic and translatomic levels, with strong positive correlations (R = 0.52–0.67). In contrast, hiSSCs displayed a decoupling of this regulation, showing a negative correlation (R = −0.11) (Fig. 3C). These findings delineate a transcription-translation decoupling mechanism in hiSSCs, highlighting the importance of cell-type-specific post-transcriptional control. Functional annotation of coordinated changes highlighted lineage-specific adaptations: hiPGCs upregulated lipid biosynthesis (*CYP27A1*, *HSD17B2*) and cytoskeletal biological process while suppressing ECM and cell migration related genes (*LAMC2*, *FN1* and *FGF1*) in the spaceflight group (Fig. 3, D and E, fig. S5, A to C). Under spaceflight conditions, hiOFs upregulated genes linked to microtubule assembly (*TUBB8*, *MAP6*) and estrogen metabolism (*CYP1A1*, *HSD17B2*), while downregulating cell adhesion-and proliferation-related networks (*COL5A1*, *COBLL1*, and *TGFBR3*) (Fig. 3, D and E, fig. S5, D to F). In contrast, hiSSC responses were largely translatome-driven (fig. S5, G and H), characterized by increased oxidative phosphorylation, translational regulation, and protein phosphorylation, together with suppression of mitotic cell cycle, cell adhesion, and actin cytoskeleton programs under spaceflight conditions (Fig. 3, D and E). This shift was consistent with the reduced hiSSC numbers observed in spaceflight (Fig. 2, G and H). Collectively, these data demonstrate that spaceflight perturbs ECM- and migration-related programs in hiPGCs and hiOFs through coordinated transcriptomic and translatomic remodeling, while preferentially reshaping the hiSSC translatome, revealing distinct cell type-specific vulnerabilities to spaceflight stress.

### Characterization of common transcriptome changes during germ cell differentiation under spaceflight conditions

**Fig. 4.**
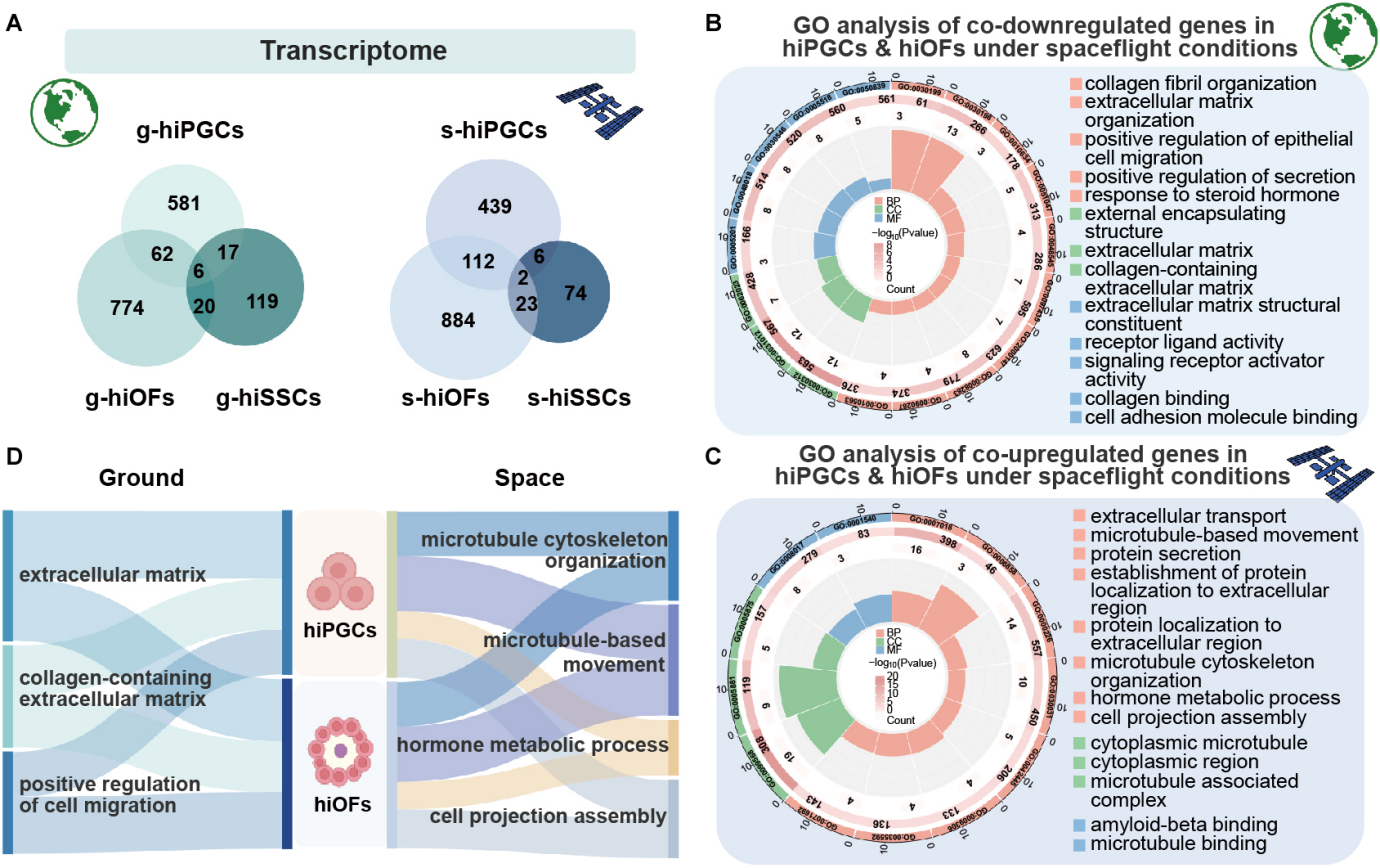
Transcriptomic changes in hiPGCs, hiOFs, and hiSSCs under spaceflight and ground-control conditions. (**A**) Venn diagrams of cell type-specific DEGs in hiPGCs, hiOFs, and hiSSCs under ground-control (left) and spaceflight (right) conditions. (**B** and **C**) GO analysis of co-up-regulated genes shared by hiPGCs and hiOFs in the transcriptome under ground-control (B) and spaceflight (C) conditions. Biological process (BP), cellular component (CC), and molecular function (MF) categories are color-coded in pink, green, and blue, respectively. Representative significant terms (*P* < 0.05), gene counts, enrichment P values, and enrichment factors are shown. (**D**) Sankey diagram of shared transcriptional regulatory effects induced by spaceflight in hiPGCs and hiOFs.

To comprehensively evaluate the broad impact of the spaceflight environment on different cell types, we performed an intercellular common differential gene analysis across three types of germ cells. Transcriptional profiling revealed that hiPGCs and hiOFs shared 114 significantly upregulated and 68 downregulated genes under spaceflight conditions, while hiSSCs exhibited minimal transcriptional changes (Fig. 4A). Enrichment analysis demonstrated that hiPGCs and hiOFs markedly downregulated genes associated with ECM organization, steroid hormone response, collagen fibril assembly, and cell adhesion, while significantly upregulating genes linked to microtubule-based motility, protein secretion, hormone metabolism, cell projection assembly, and amyloid-beta binding (Fig. 4, B to D). Collectively, these results demonstrate a shared transcriptional response in hiPGCs and hiOFs, characterized by coordinated dysregulation of pathways related to ECM and cytoskeletal organization, while hiSSCs maintained transcriptional stability, indicating a lineage-specific pattern of gene regulation under spaceflight conditions.

### Characterization of common translatome changes during germ cell differentiation under spaceflight conditions

**Fig. 5.**
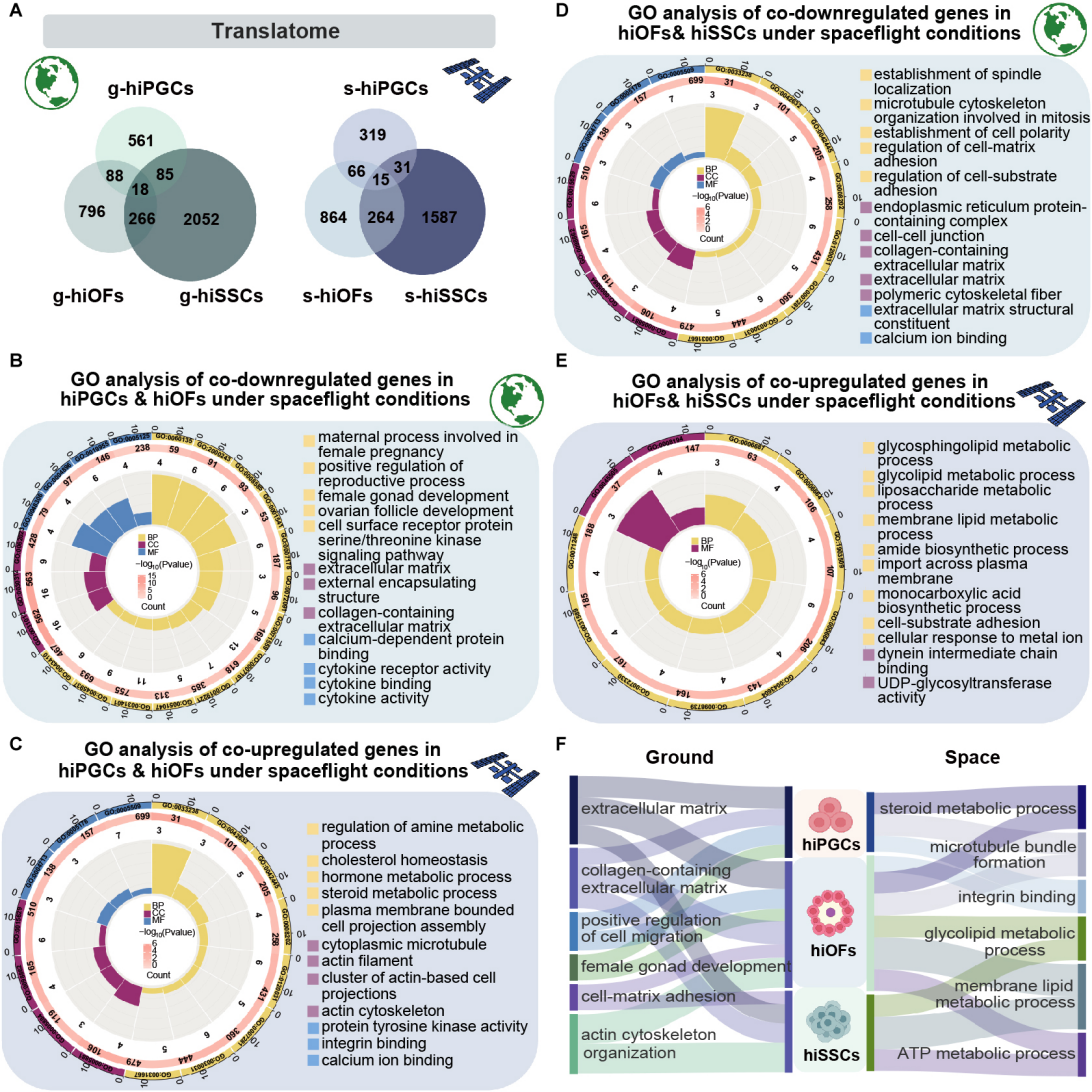
Common translatome alterations across cell types under spaceflight conditions. (**A**) Venn diagram of the DEGs (FDR < 0.05, |log2FC| ≥1) in the translatome profiles of hiPGCs, hiOFs, and hiSSCs under ground-control (left) and spaceflight (right) conditions. (**B** to **E**) GO analysis of shared translationally up-regulated genes across cell types: hiPGC-hiOF overlap genes under ground-control conditions (B), hiPGC-hiOF overlap genes under spaceflight conditions (C), hiOF-hiSSC overlap genes under ground-control conditions (D), and hiOF-hiSSC overlap genes under spaceflight conditions (E). BP, CC, and MF categories are color-coded in yellow, purple, and blue, respectively. Detailed annotations for each concentric-circle layer are provided in Fig. 4. Representative significant terms (*P* < 0.05) are shown. (**F**) Sankey diagram of pathways associated with conserved spaceflight-induced translatome perturbations across hiPGCs, hiOFs, and hiSSCs.

Given the critical role of post-transcriptional regulation of mRNAs in germ cell development (*33*, *34*, *39*–*41*), we analyzed common translational changes across the three germ cell types under spaceflight conditions. Translational profiling showed that hiPGCs and hiOFs shared 81 upregulated and 106 downregulated genes, whereas hiOFs and hiSSCs exhibited 279 upregulated and 284 downregulated targets. In contrast, hiPGCs and hiSSCs displayed fewer translational alterations (46 upregulated, 103 downregulated), which were insufficient for robust functional enrichment analysis (Fig. 5A). GO analysis mapped the translationally downregulated genes in hiPGCs and hiOFs to ECM components, calcium-dependent protein binding, cytokine receptor activity, and female germ cell developmental processes, including maternal pregnancy regulation, ovarian follicle development, and reproductive system modulation (Fig. 5B). These alterations reflect adaptive responses to disrupted cytoskeletal dynamics and energy metabolism under spaceflight conditions, aligning with the observed reduction in hiPGC differentiation efficiency under spaceflight conditions (Fig. 2, B to E). In contrast, spaceflight induced translational upregulation of genes governing amine metabolism, cholesterol homeostasis, steroid metabolism, cytoplasmic microtubule organization, actin cytoskeleton dynamics, integrin binding, and calcium signaling (Fig. 5C). Together, these data show that spaceflight induces a coordinated response at the translational level in hiPGCs and hiOFs: downregulation of female germline developmental programs, alongside upregulation of cytoskeletal and metabolic pathways. The combined analysis of hiOFs and hiSSCs revealed conserved translational patterns, including downregulation of ECM-related genes and upregulation of metabolic regulators. Specifically, pathways regulating microtubule organization during mitosis, cell polarity determination, and cell-matrix adhesion exhibited suppressed translational activity in space-exposed samples compared to ground controls (Fig. 5D), while glycosphingolipid metabolism, glycolipid processing, membrane lipid homeostasis, and amide biosynthesis pathways showed enhanced translational activity under spaceflight conditions (Fig. 5E). These findings reveal a coordinated translational alteration toward structural remodeling and lipid metabolism in hiOFs and hiSSCs during spaceflight.

Integrated analysis identified ECM disruption and collagen network alterations as common spaceflight effects across three different germ cell types. hiPGCs and hiOFs exhibited reduced translation of cell migration and female gonad development genes during spaceflight, while hiOFs and hiSSCs showed suppressed translation of adhesion- and actin cytoskeleton-related transcripts. Conversely, spaceflight-exposed germ cells translationally upregulated pathways governing microtubule bundle formation, integrin-mediated ATP metabolism, steroidogenesis, and membrane lipid metabolism (Fig. 5F). Overall, the conserved translational modulation in ECM, cytoskeletal, and lipid metabolic pathways across germ cell models indicates a unified adaptive response to spaceflight, underscoring the importance of post-transcriptional regulation in mediating human germ cell vulnerability to the spaceflight environment.

### Preserved genomic integrity and methylome stability under low-dose space radiation

During the 6- to 15-day TZ-6 mission, onboard dosimeters recorded a cumulative radiation dose of 1.66 to 4.14 mGy. This standard low-Earth orbit exposure, primarily comprising galactic cosmic rays and trapped radiation in the South Atlantic Anomaly (*42*), is markedly lower than the prolonged exposures known to induce genomic instability and chromosomal rearrangements (*51*, *52*).

**Fig. 6.**
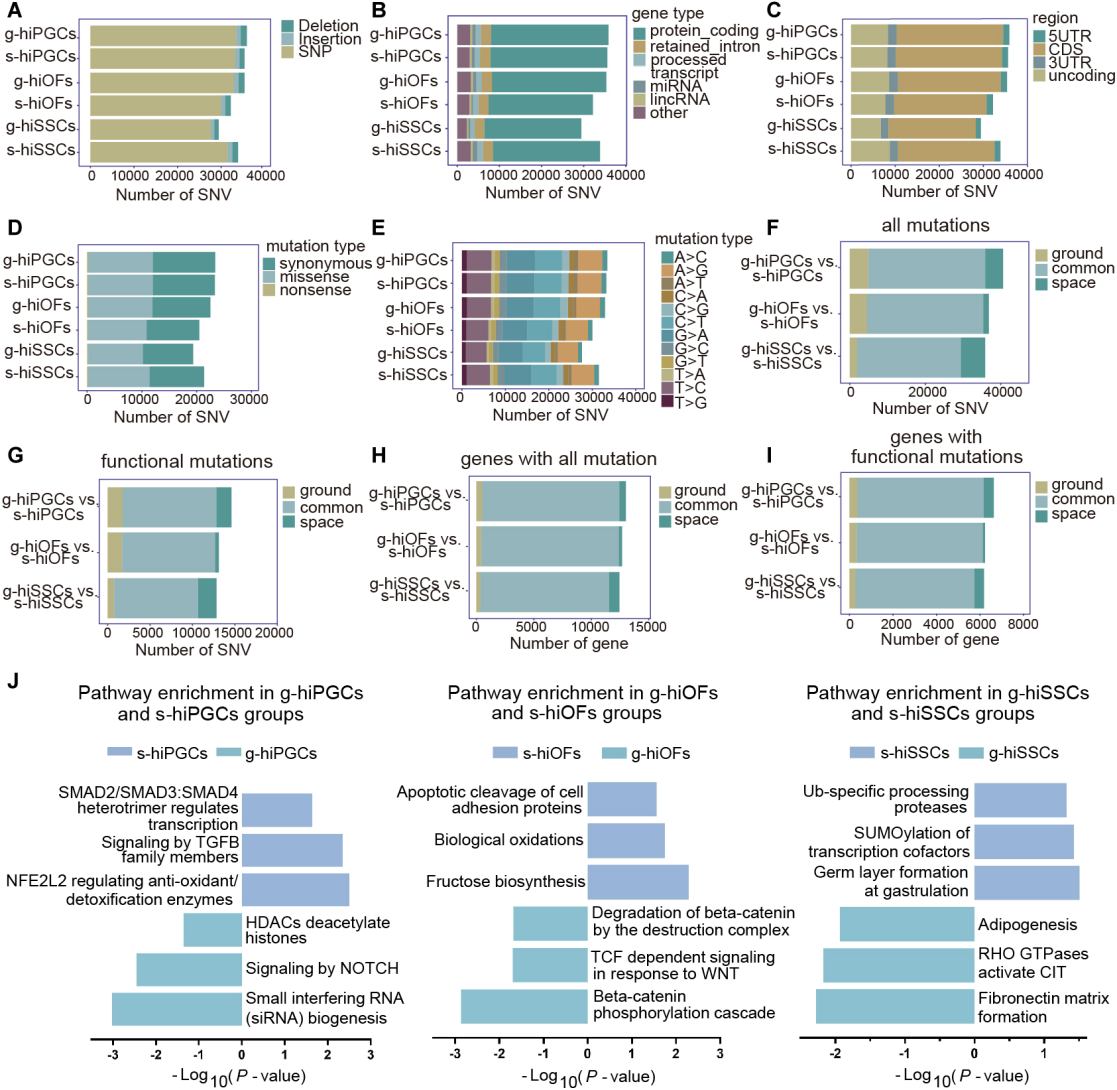
Mutational landscape across cell types under spaceflight and ground-control conditions. (**A**) Distribution of mutation types in hiPGCs, hiOFs, and hiSSCs under ground-control and spaceflight conditions. (**B**) Distribution of mutated gene categories between the spaceflight and ground-control groups across the three cell types. (**C**) Genomic distribution of mutated genes in hiPGCs, hiOFs, and hiSSCs under both conditions. (**D**) Proportions of synonymous, missense, and nonsense mutations. (**E**) Base-substitution patterns across cell types and experimental conditions. (**F**) Comparison of the numbers of SNVs among the three cell types under spaceflight and ground-control conditions. (**G**) Proportion of genes harboring functional mutations in the spaceflight and ground-control groups. (**H**) Comparison of the numbers of genes harboring any mutations among the three cell types under the two conditions. (**I**) Number of genes harboring functional mutations across cell types and conditions. (**J**) Reactome pathway enrichment analysis of missense-mutated genes in hiPGCs, hiOFs, and hiSSCs under ground-control and spaceflight conditions.

To determine whether the spaceflight environment compromises genomic integrity during hESC differentiation, we performed whole-exome sequencing (WES) on both spaceflight-cultured and ground-control samples. Global mutational parameters, including the frequency of deletions, insertions, and single-nucleotide polymorphisms (SNPs), showed no appreciable differences across experimental groups (Fig. 6A). Furthermore, variant distributions across gene categories and genomic positions, as well as base-substitution signatures, exhibited high consistency between spaceflight and ground-control conditions (Fig. 6, B to E). Similarly, hiPGCs and hiOFs showed no significant differences in either the number of single-nucleotide variants (SNVs) or the number of genes harboring mutations (Fig. 6, F to I). In contrast, the hiSSC lineage in the spaceflight group exhibited a significant increase in mutational burden, characterized by a higher number of SNVs and mutated genes relative to ground controls. Notably, spaceflight-exposed hiSSCs exhibited a 3.3-fold increase in genes harboring all mutations and a 2.8-fold increase in genes carrying functional mutations relative to ground-control conditions (Fig. 6, F and G). This difference likely reflects the longer in-orbit differentiation period for hiSSCs (15 days) than for hiPGCs and hiOFs (6 days), suggesting that genomic damage in human germ cells may accumulate with prolonged orbital exposure.

To evaluate the functional implications of these identified variants (table S1), we performed Reactome pathway enrichment analysis on genes harboring missense mutations (Fig. 6J). Mutational landscapes diverged by environmental condition across all lineages: in hiPGCs, spaceflight variants targeted NFE2L2-mediated antioxidant responses, whereas ground-control mutations concentrated on small interfering RNA (siRNA) biogenesis; in hiOFs, spaceflight mutations enriched fructose biosynthesis pathways, contrasting with WNT signaling in ground controls; and in hiSSCs, spaceflight variants mapped to germ layer formation rather than the fibronectin matrix assembly implicated under ground-control conditions.

Crucially, these mutational signatures exhibited minimal concordance with the coordinated transcriptomic and translational remodeling occurring across all three germ cell lineages (Figs. 3 to 5). This decoupling indicates that the mutational profiles detected via WES do not drive the acute spaceflight-induced molecular remodeling; rather, this remodeling represents a plastic adaptive response to environmental stressors. Corroborating this, whole-genome bisulfite sequencing (WGBS) confirmed that the cells preserve their global DNA methylation patterns during spaceflight, despite subtle lineage-specific variations (fig. S6). Collectively, our data demonstrate that short-term spaceflight does not substantially compromise the genomic or epigenomic stability of human germ cell models.

## DISCUSSION

As humanity advances toward deep space exploration, understanding the effects of the spaceflight environment on human reproductive biology has become an important frontier in space medicine. Emerging evidence from rodent studies reveals microgravity-induced seminiferous tubule degeneration in male mice (*53*, *54*), disrupted estrous cycles in female rats (*55*) and impaired follicular development in vitro (*56*). However, the mechanistic insights remain poorly defined. Given the ethical constraints on human germ cell experimentation, pluripotent stem cell-derived in vitro models provide a tractable platform for investigating developmental vulnerabilities of the human germline in space.

In this study, we integrate live-cell imaging and multi-omics profiling to evaluate the spaceflight-induced differentiation of three hESC-derived human germ cell models: hiPGCs, hiOFs, and hiSSCs. Real-time imaging revealed a spaceflight-induced reduction in VASA-GFP^+^ hiPGCs with diminished fluorescence intensity and suppressed hiSSC proliferation, whereas hiOFs retained grossly normal morphology under spaceflight conditions. Despite morphological stability in certain lineages, multi-omic profiling uncovered profound molecular perturbations; specifically, hiOFs underwent coordinated transcriptome-translatome remodeling of ECM and cytoskeletal programs, whereas hiSSCs exhibited pronounced translatome-level reprogramming. These molecular signatures parallel ECM remodeling observed in astronaut muscle biopsies (*8*) and *Drosophila* cardiomyocytes (*18*), suggesting an evolutionarily conserved mechanobiological response to microgravity. Although our models capture prenatal germ cell development, this developmental window is highly relevant to future space medicine. As long-duration missions increase the prospect of pregnancy in space, prenatal germ cells emerge as a critical vulnerability in reproductive risk assessment. Given the critical role of extracellular matrix organization and cytoskeletal integrity in dictating adult reproductive architecture, such as the seminiferous tubule basement membrane and ovarian follicles, early developmental disruption of these structural foundations poses profound long-term risks. Consequently, our findings establish a mechanistic framework for spaceflight-induced gonadal degeneration, highlighting the extreme susceptibility of early germ cell programming. Future studies are required to elucidate how these prenatal anomalies impact adult reproductive physiology and long-term fertility.

Additionally, translational profiling uncovered a concerted upregulation of glycolipid and membrane lipid metabolic pathways in both hiOFs and hiSSCs. This metabolic shift is consistent with the broader lipid dysregulation observed during spaceflight, including hepatic lipid accumulation in murine models (*57*, *58*) and altered plasma lipid-associated proteomic signatures in astronauts (*59*). Together, these data suggest that developing human germ cells undergo a coupled structural and metabolic adaptation to spaceflight stress, which provide a mechanistic basis for future studies in adult-relevant models, gonadal niche systems, and longer-exposure platforms to evaluate the potential significance of these responses for reproductive health.

In contrast to these transcriptomic and translational changes, our genomic and epigenomic analyses revealed remarkable stability. Early germ cell development is a highly dynamic and environmentally sensitive stage characterized by extensive molecular reprogramming. In this context, our findings suggest that spaceflight-associated stress preferentially perturbs transcriptomic, translatomic, and structural regulatory programs during early developmental progression. WES detected limited mutational divergence, and WGBS showed largely preserved DNA methylation patterns during this short-term mission, indicating that broad genomic or epigenomic destabilization was not evident within the current exposure window. The contrast between rapid functional remodeling and limited genomic damage suggests that short-term spaceflight primarily elicits an adaptive cellular response rather than overt genotoxic collapse. At the same time, the increased mutational burden observed in hiSSCs after the longer 15-day exposure raises the possibility that duration-dependent damage may emerge with more prolonged orbital residence.

A limitation of this study is the restricted number of biological replicates dictated by inherent spaceflight payload constraints. To maximize analytical robustness within this operational framework, we quantified multi-field imaging data. Although in-orbit fixation precluded post-flight assessment of proliferation markers such as Ki-67 and BrdU post-flight, our 15-day time-lapse imaging dataset provided a longitudinal readout of cell growth and survival. Future studies incorporating longer exposure durations and serial in-orbit sampling will refine the temporal profile of germ cell adaptation to spaceflight. The integration of automated in-orbit staining with orthogonal functional readouts would further strengthen mechanistic inference and inform evidence-based countermeasures for reproductive health during long-duration space missions. From a translational perspective, our findings suggest several directions for future risk-mitigation strategies, including preflight gamete cryopreservation, in-flight monitoring of reproductive and stress-related biomarkers, and mechanism-informed approaches to preserve reproductive niche integrity through maintenance of ECM organization, cytoskeletal stability, and metabolic homeostasis. Although future studies should validate these approaches, our molecular and structural readouts provide a crucial foundation for assessing reproductive risks and designing targeted countermeasures. Moreover, in the absence of post-flight follow-up in the current study design, future missions could incorporate serial sampling during the recovery phase to determine the persistence and reversibility of these spaceflight-induced germ cell alterations.

Overall, our study identifies ECM, cytoskeletal, and translational remodeling as central and conserved features of the human germ cell response to spaceflight. These findings provide a framework for examining early human germ cell developmental responses in space and nominate candidate pathways for further mechanistic investigation. Future studies in extended on-orbit systems, human organoids and adult-relevant models will be needed to determine the broader significance of these responses for reproductive biology.

## MATERIALS AND METHODS

### Cell culture

The hESC line H9 (female XX line) was purchased from WiCell, Inc., and propagated as described previously (*60*). In brief, undifferentiated hESCs were expanded on irradiated mouse embryonic fibroblast feeder layers in KnockOut DMEM (Gibco, 10829018) supplemented with 20% KnockOut Serum Replacement (Gibco, A3181502), 25 μg/ml recombinant human basic fibroblast growth factor (R&D, 233-FB/CF), 1% penicillin-streptomycin (Gibco, 15140163), 1 mM GlutaMAX (Gibco, 35050061), and 1% NEAA (Gibco, 11140076).

The hESC line H1 (male XY line) was purchased from WiCell, Inc., and cultured on Matrigel-coated plates (Corning, 365230) using E8 medium (Gibco, A1517001) with 1% penicillin-streptomycin. Cultures were maintained at 37°C in a humidified atmosphere with 5% CO2. For the subculture, hESCs were rinsed twice with DPBS (Yeasen, 60152ES76), and detached as single cells with EDTA (Cellapy, CA1023100) at 37°C for 2 min and replated on Matrigel-coated plates. The fresh medium was changed daily, and cells were passaged every four days.

To differentiate H9 cell line into hiPGCs, cells were seeded onto Matrigel-coated (Corning, 354248) cell culture charmber at 30–40% confluency and propagated for 24 hours. Differentiation was initiated using PGC-specific medium containing KnockOut DMEM supplemented with 10% fetal bovine serum (FBS, Gibco, 10099141), 1 mM GlutaMAX, 1% NEAA, 1% penicillin-streptomycin, and 50 ng/ml recombinant human BMP4 (Proteintech, HZ1045). Cells were maintained at 37°C in a humidified atmosphere with 5% CO_2_, with complete medium replacement every 48 hours.

hESCs (H9) were differentiated into hiOFs following an established protocol (*31*) with modifications. Briefly, 5 × 10^4^ undifferentiated hESCs (50% confluency) were seeded onto Matrigel-coated six-well plates. Differentiation was initiated by treating cells with induction medium (KnockOut DMEM supplemented with 10% FBS, 1 mM GlutaMAX, 1% NEAA, and 50 ng/ml BMP4 and BMP8a (R&D, 1073-BPC) for 1 h at 37°C with 5% CO_2_. Cells were then transduced with lentiviral vectors expressing DAZL and BOLL for 24 hours, followed by 24 hours recovery and 72 hours blasticidin selection (2 μg/ml). At day 7, cells were treated with differentiation medium containing 5 ng/ml GDF9:BMP15 heterodimer [purified according to established protocol (*61*)] and 10 ng/ml EGF (R&D Systems) for 24 hours. Subsequent differentiation medium containing 50 ng/ml GDF9 and 25 ng/ml BMP15 was refreshed every 48 hours.

For hiSSC differentiation, H1 hESCs were seeded onto Matrigel-coated culture vessels at 30–40% confluency and propagated for 48 hours. Differentiation was initiated using SSC-specific medium containing MEMα (Invitrogen) supplemented with 3% KnockOut Serum Replacement (Invitrogen), 1 mM GlutaMAX, 1× ITS-X (Invitrogen), 0.2 mg/ml vitamin C (Sigma), 1× Chemically Defined Lipid Concentrate (Invitrogen), 1 ng/ml bFGF, and 20 ng/ml recombinant human GDNF (Abclonal). Medium was refreshed every 36 hours.

### Sample processing

Post-flight cell samples from both spaceflight and ground control groups were preserved in RNALater (Beyotime, R0118) and stored at −80°C. Upon return to the laboratory, residual RNALater was removed through three PBS washes. Subsets of cells were allocated for T&T-seq, while genomic DNA was extracted using TRIzol Reagent (Invitrogen) for WES and WGBS.

### Transcriptome-translatome sequencing

Transcriptome-translatome sequencing followed Hu *et al*. with modifications (*37*). Briefly, cell lysates were partitioned into transcriptome (2 μl) and translatome (8 μl) fractions. Ribosome-bound mRNAs were captured using RiboLace beads (Immagina) through 70-min rotational incubation at 4°C. Post-capture beads underwent two washes with W-buffer (Immagina), followed by ribosome-mRNA complex dissociation using 1% SDS and proteinase K treatment (37°C, 30 min). RNA was purified via TRIzol/chloroform extraction and precipitated overnight.

Full-length cDNA synthesis was performed using the Single Cell Full-Length mRNA-Amplification Kit (Vazyme). Libraries were constructed with the TruePrep DNA Library Prep Kit V2 (Vazyme) and sequenced on the NovaSeq 6000 platform (Illumina) to achieve >20 million 150-bp paired-end reads per sample.

### Library preparation for WGBS

Genomic DNA was subjected to bisulfite conversion using the EpiArt DNA Methylation Bisulfite Kit (Vazyme) under the following thermal cycling conditions: 98°C for 10 minutes, followed by 64°C for 40 minutes (3 cycles). Bisulfite-converted DNA was purified using spin columns and subsequently processed with the VAHTS Universal Pro DNA Library Prep Kit (Vazyme). Libraries were quantified using Qubit fluorometry and Agilent Bioanalyzer, followed by 150-bp paired-end sequencing on the Illumina NovaSeq 6000 platform.

### Library preparation for WES

Genomic DNA (200 ng) was fragmented to less than 300 bp using a Covaris S220 ultrasonicator. Fragmented DNA was subjected to end repair (including 5′ phosphorylation and 3′ adenylation) and adapter ligation using the End Prep Enzyme Mix. Fragments of approximately 300 bp were size-selected using magnetic beads, with insert sizes ranging from 200 to 250 bp. Libraries were amplified using P5 and P7 primers, followed by purification with magnetic beads. For hybridization capture, 750 ng of the library was mixed with probes, hybridization buffer, and blockers, and incubated for no more than 24 hours. Non-specifically bound products were removed by washing, and the enriched library was amplified for final library preparation. Quality control was performed prior to sequencing. Indexed libraries were pooled and sequenced using the Illumina NovaSeq platform with 150-bp paired-end reads, following the manufacturer’s instructions.

### Bioinformatics analysis

#### T&T-seq data processing

Raw sequencing reads were processed through a standardized bioinformatics pipeline. Quality control and adapter trimming were performed with Trim Galore (v0.6.4). Clean reads were aligned to the human reference genome (GRCh38/hg38; GENCODE release v42) using HISAT2 (v2.1.0) (*62*) and gene-level read counts were quantified with featureCounts (v1.6.5) (*63*) based on GENCODE gene annotation in GTF format. Transcript abundance was normalized to transcripts per million (TPM) using an in-house Python script. Differential expression analysis was performed with DESeq2 (v1.26.0) (*64*). PCA was performed in DESeq2 using genes with counts greater than 10 in at least half of the samples. Gene Ontology (GO) enrichment analysis was conducted using the Metascape web tool with default parameters (minimum overlap = 3, *P* value cutoff = 0.01, and minimum enrichment = 1.5). KEGG pathway enrichment analysis of differentially expressed genes was performed using KOBAS v3.0, with significance thresholds of *P* < 0.05 and fold change ≥1.5. GSEA was performed using the Broad Institute GSEA software with default settings, including phenotype permutation, 1000 permutations, weighted enrichment statistics, and minimum and maximum gene set sizes of 15 and 500, respectively. Pathways with *P* < 0.05 were considered significantly enriched. Heatmaps were generated using TBtools (v1.09854).

#### WGBS data processing

Raw sequencing reads were trimmed by 9 nucleotides, and low-quality reads and adapters were removed using Trim Galore (v0.6.10). Cleaned reads were aligned to the in silico bisulfite-converted human reference genome (hg38) using Bismark (v0.24.1) with non-directional mapping parameters. Duplicate reads were removed using the deduplicate_bismark command. Methylation levels at single CpG sites were extracted using bismark_methylation_extractor. CpG sites with at least one read coverage were retained for downstream analysis. Methylation levels were calculated as the ratio of methylated cytosines (C) to total cytosines (C + T) at each CpG site. Global DNA methylation levels were calculated by binning the genome into 1 Mb tiles, with methylation levels computed as the total methylated counts divided by the total counts within each bin. Only tiles with coverage of at least five CpG sites were included.

#### WES data processing

Raw sequencing data were preprocessed using fastp (v0.23.4) (*65*) to remove adapter sequences. Clean reads were aligned to the human reference genome (hg38) using the Burrows-Wheeler Aligner (BWA, version 0.7.17-r1188) (*66*). Base quality score recalibration, indel realignment, and duplicate removal were performed using the Genome Analysis Toolkit (GATK) (*67*). Variant calling, including single nucleotide polymorphisms (SNPs) and insertions/deletions (INDELs), was conducted across all samples using GATK Best Practices recommendations, including standard hard filtering parameters or variant quality score recalibration. All SNVs and indels were subsequently annotated using Ensembl Variant Effect Predictor (VEP) to assign functional consequences at the gene and transcript levels based on the hg38 reference annotation. Genes carrying missense variants were used for downstream Reactome pathway enrichment analysis.

### Image analysis

Fluorescence images were processed using OpenCV (version 4.8.0; OpenCV Foundation, 2023) for background subtraction and contrast enhancement. The Segment Anything Model (SAM) (*68*) was implemented for fluorescence segmentation, while Cellpose (*69*) was adapted for brightfield cell counting. All analyses were performed on raw TIFF files to preserve spatial resolution.

### Statistical analysis

The cell area and fluorescence intensity of GFP - positive cells were analyzed using GraphPad Prism 7 (GraphPad Software, La Jolla, CA). Because of payload and operational constraints during the TZ-6 mission, each condition included two independent culture samples, which were considered biological replicates. For imaging-based analyses, multiple non-overlapping fields were acquired from each biological replicate at each time point and analyzed. Unless otherwise stated, N refers to the number of image fields analyzed. Data are presented as mean ± SD. Statistical significance was assessed using Student’s t-test. Differences were considered statistically significant at *P* < 0.05 and are indicated as follows: *P < 0.05, **P < 0.01, ***P < 0.001 and ****P < 0.0001.
