# Supplementary material for: Spaceflight multi-omics reveals vulnerabilities of human germ cell development
Source: Sci Adv. 2026 Jul 15;12(29):eaee0143. doi: 10.1126/sciadv.aee0143 (PMC13371913; doi:10.1126/sciadv.aee0143)
Supplement: Supplementary file 1 [file sciadv.aee0143_sm.pdf]

Science Advances
AAAS

Supplementary Materials for

Spaceflight multi-omics reveals vulnerabilities of human germ cell development

Ying Li *et al.*

Corresponding author: Ying Li, liying.2018@tsinghua.org.cn; Qiangfeng Cliff Zhang, qczhang@tsinghua.edu.cn; Tao Zhang, haozzh@sina.com; Kehkooi Kee, kkee@tsinghua.edu.cn

Sci. Adv. **12**, eaee0143 (2026)
DOI: 10.1126/sciadv.aee0143

The PDF file includes:

Figs. S1 to S6
Legend for table S1

Other Supplementary Material for this manuscript includes the following:

Table S1

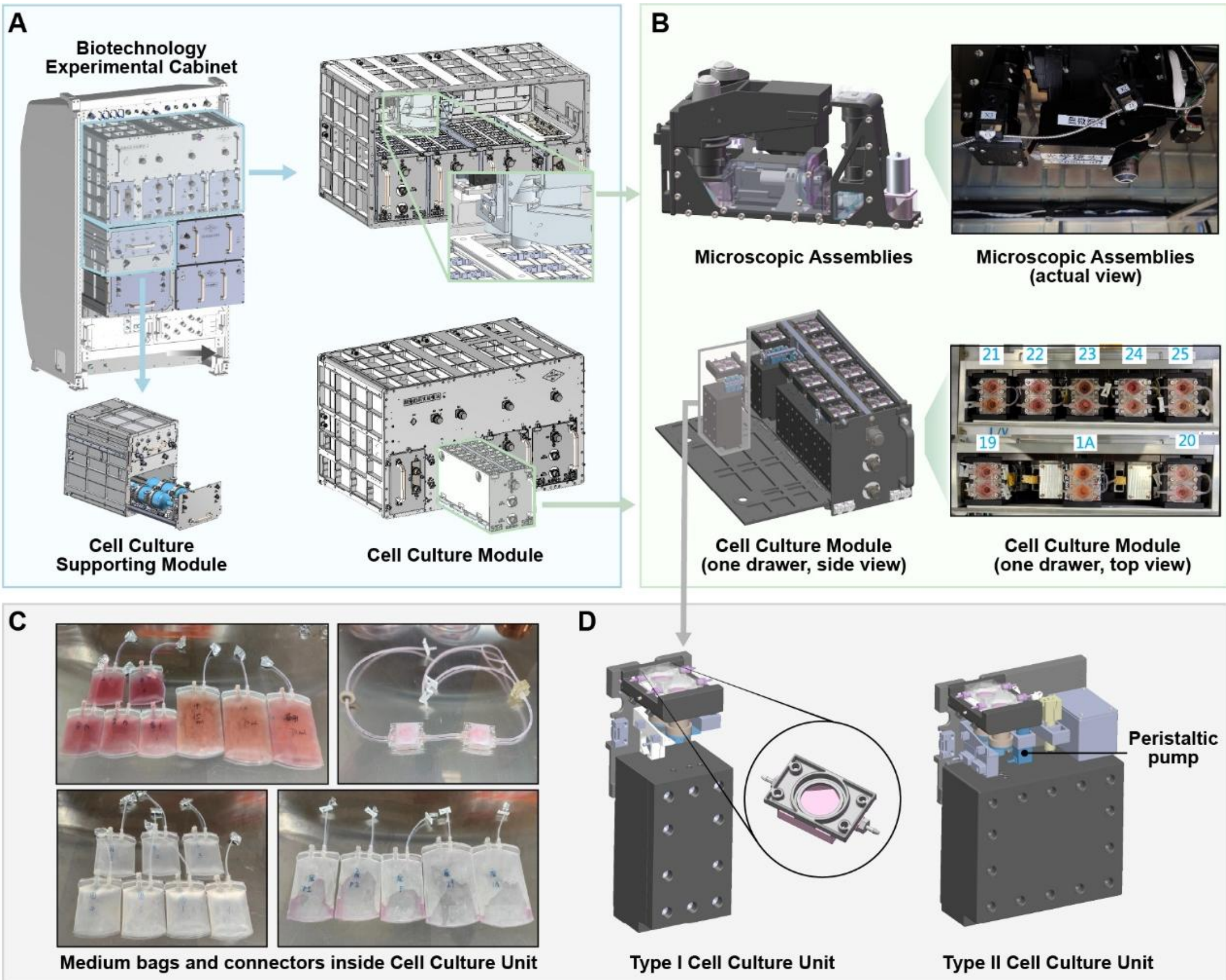

Fig. S1. Automated culture platform for the generation and collection of hESC-derived germ cell-like cells.

(A) Bioreactor modules of the biotechnology cabinet, including the cell culture support and culture modules. (B) Integrated microscopic imaging assembly for real-time monitoring of cellular morphology. (C) Medium bags and fluidic connectors within the culture unit. Upper left, culture medium bag; upper right, culture chamber; lower left, nucleic acid preservation solution bag; lower right, waste bag. (D) Fully assembled culture unit with the culture chamber and modular fluid reservoirs for microgravity-compatible perfusion.

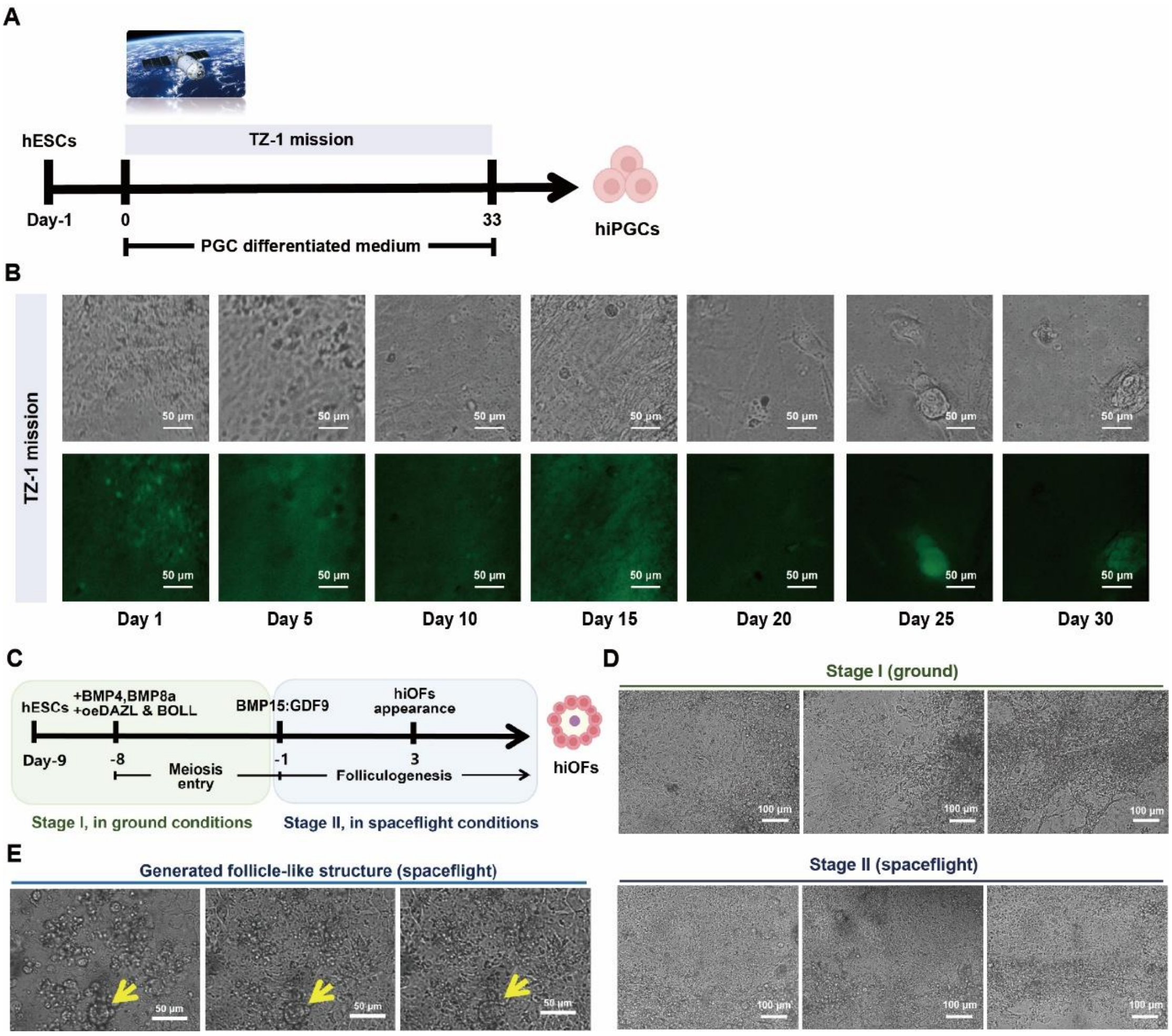

Fig. S2. Morphological characterization of hiPGC and hiOF differentiation under spaceflight conditions.

(A) Schematic of the 30-day hiPGC differentiation protocol in the TZ-1 mission. (B) Fluorescence images of DDX4 expression and cellular morphology in hiPGCs under spaceflight conditions at day 30 of differentiation in the TZ-1 mission. (C) Schematic of the 6-day hiOF differentiation protocol in the TZ-6 mission. (D) Bright-field images of hiOF differentiation at two stages under ground-based (stage I) and spaceflight (stage II) conditions. Scale bars, 100 µm. (E) Follicle-like structures under spaceflight conditions. Scale bars, 50 µm. Yellow arrows, positions of hiOFs.

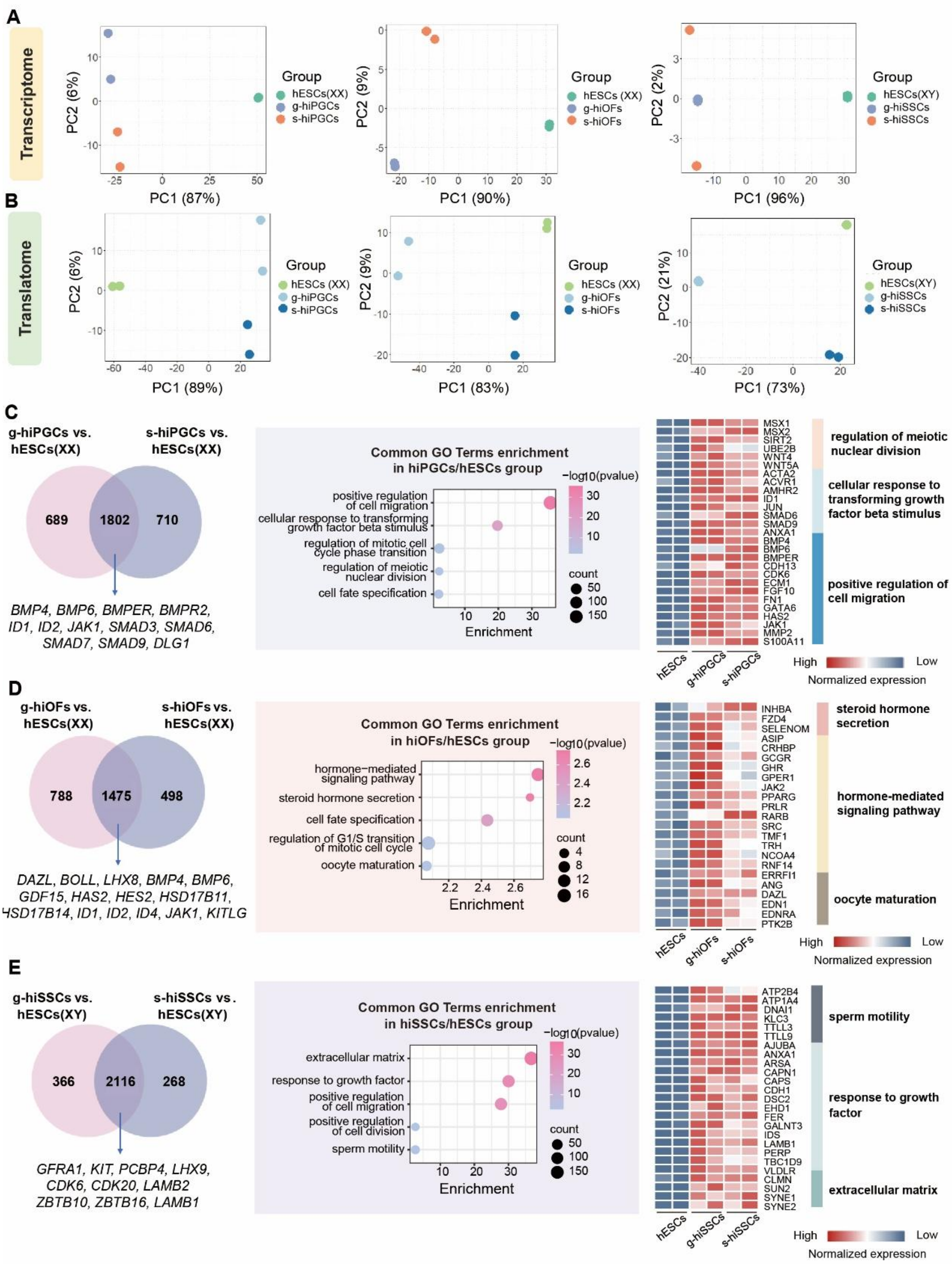

Fig. S3. Transcriptomic and translatomic features of induced germ cell differentiation under spaceflight and ground-control conditions.

(A, B) PCA plots of transcriptomic (A) and translatomic (B) profiles in spaceflight and ground groups relative to hESCs across hiPGCs (left), hiOFs (middle), and hiSSCs (right). (C to E) Venn diagrams (left) of genes specifically expressed in hiPGCs (C), hiOFs (D), and hiSSCs (E) under spaceflight and ground-control conditions relative to hESCs, GO enrichment analysis (middle) of genes shared between the two conditions, and heatmaps (right) of representative genes.

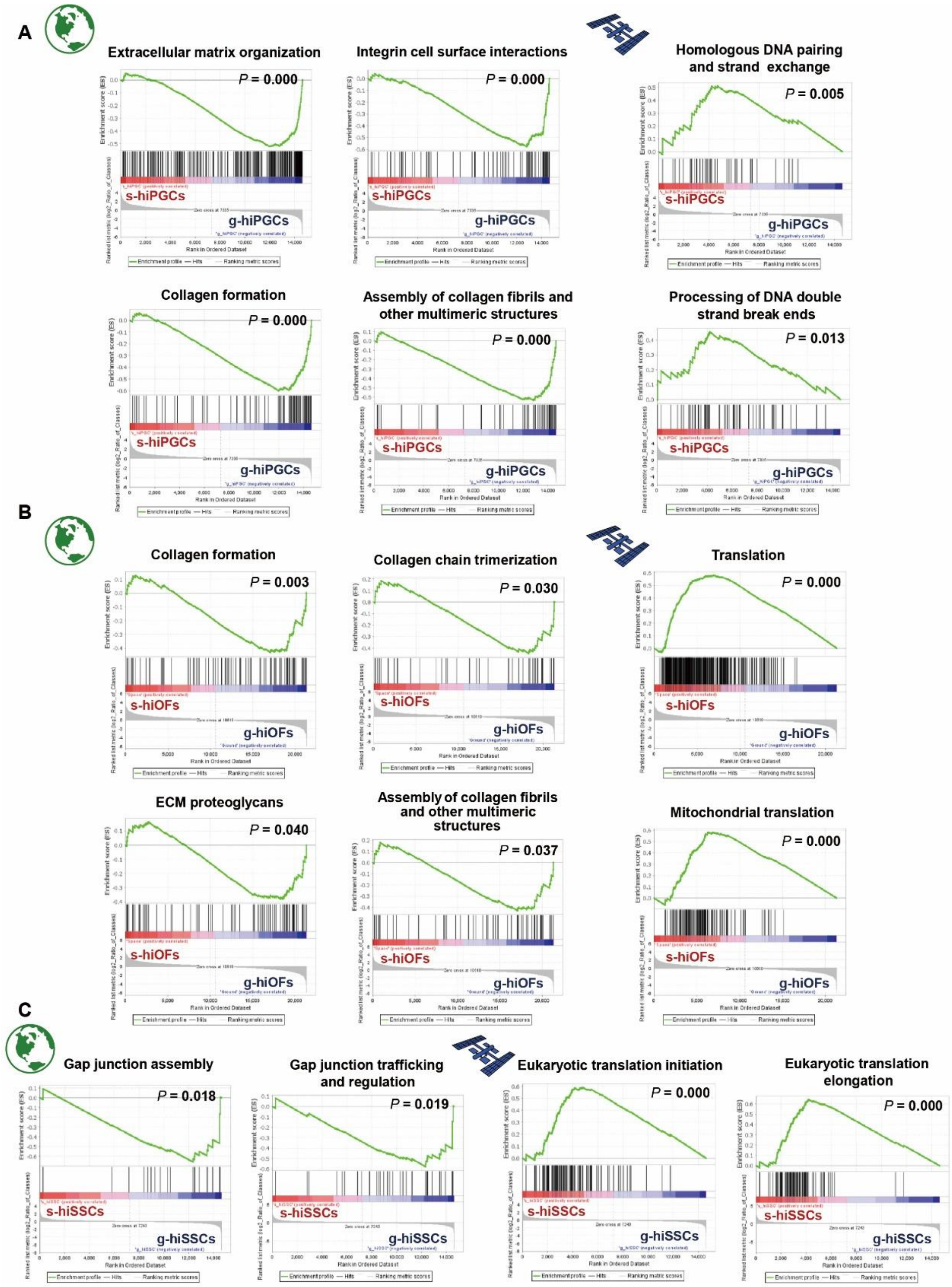

Fig. S4. GSEA/ Reactome analysis of transcriptomic differences across three germ cell types under spaceflight and ground-control conditions.

(A-C) GSEA/Reactome analysis of hiPGCs (A), hiOFs (B), and hiSSCs (C) under spaceflight and ground-control conditions.

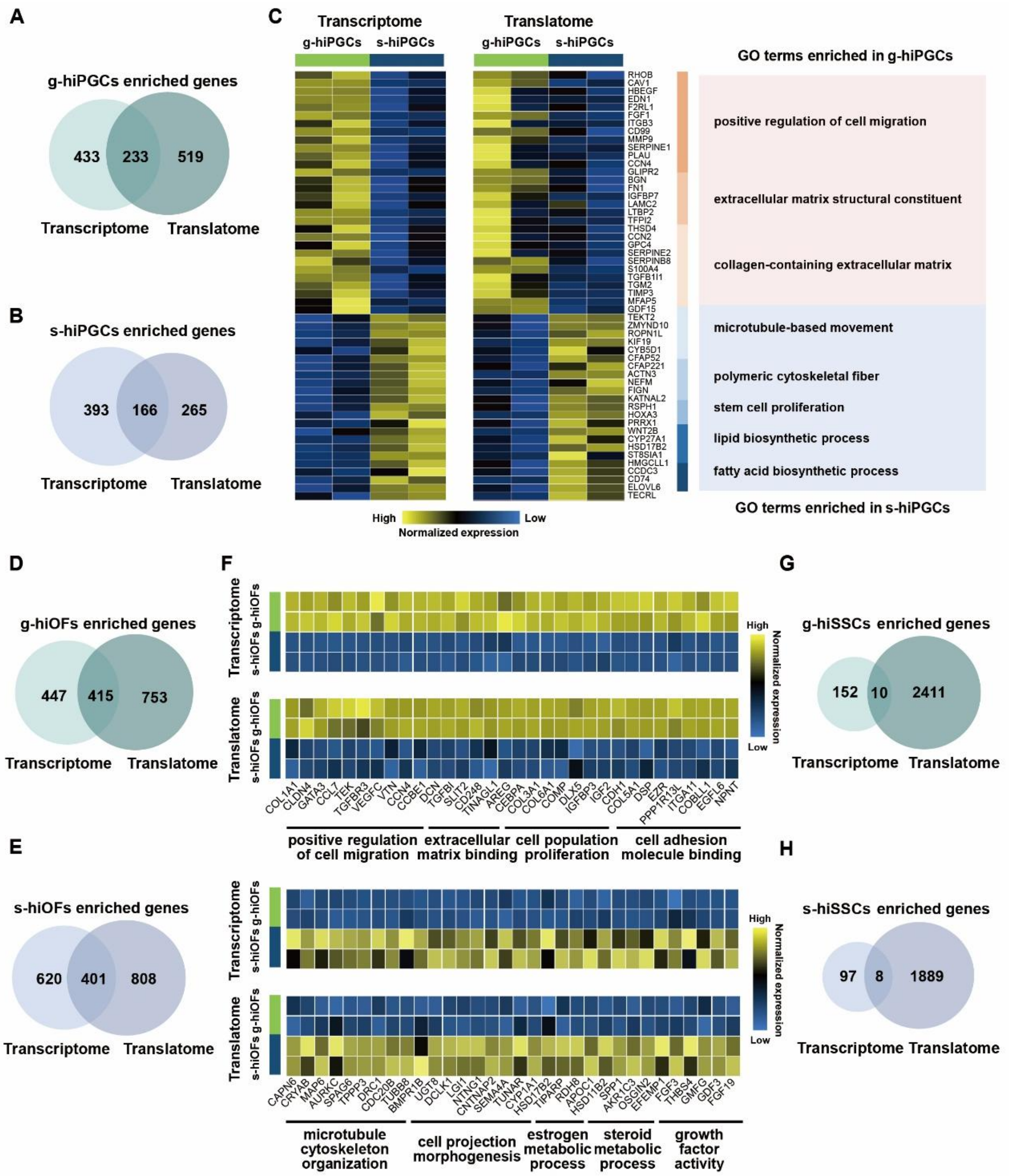

Fig. S5. Concordant transcriptomic and translatomic responses to spaceflight in hESC-derived germ cell-like cells.

(A, B) Venn diagrams of enriched genes in the transcriptome and translatome of hiPGCs under ground-control (A) and spaceflight (B) conditions. (C) Heatmap and GO analysis of shared DEGs identified in the transcriptome and translatome of hiPGCs under ground-control and spaceflight conditions. (D, E) Venn diagrams of enriched genes in the transcriptome and translatome of hiOFs under ground-control (D) and spaceflight (E) conditions. (F) Heatmap and GO analysis of shared DEGs identified in the transcriptome and translatome of hiOFs under ground-control (up) and spaceflight (down) conditions. (G, H) Venn diagrams of enriched genes in the transcriptome and translatome of hiSSCs under ground-control (G) and spaceflight (H) conditions .

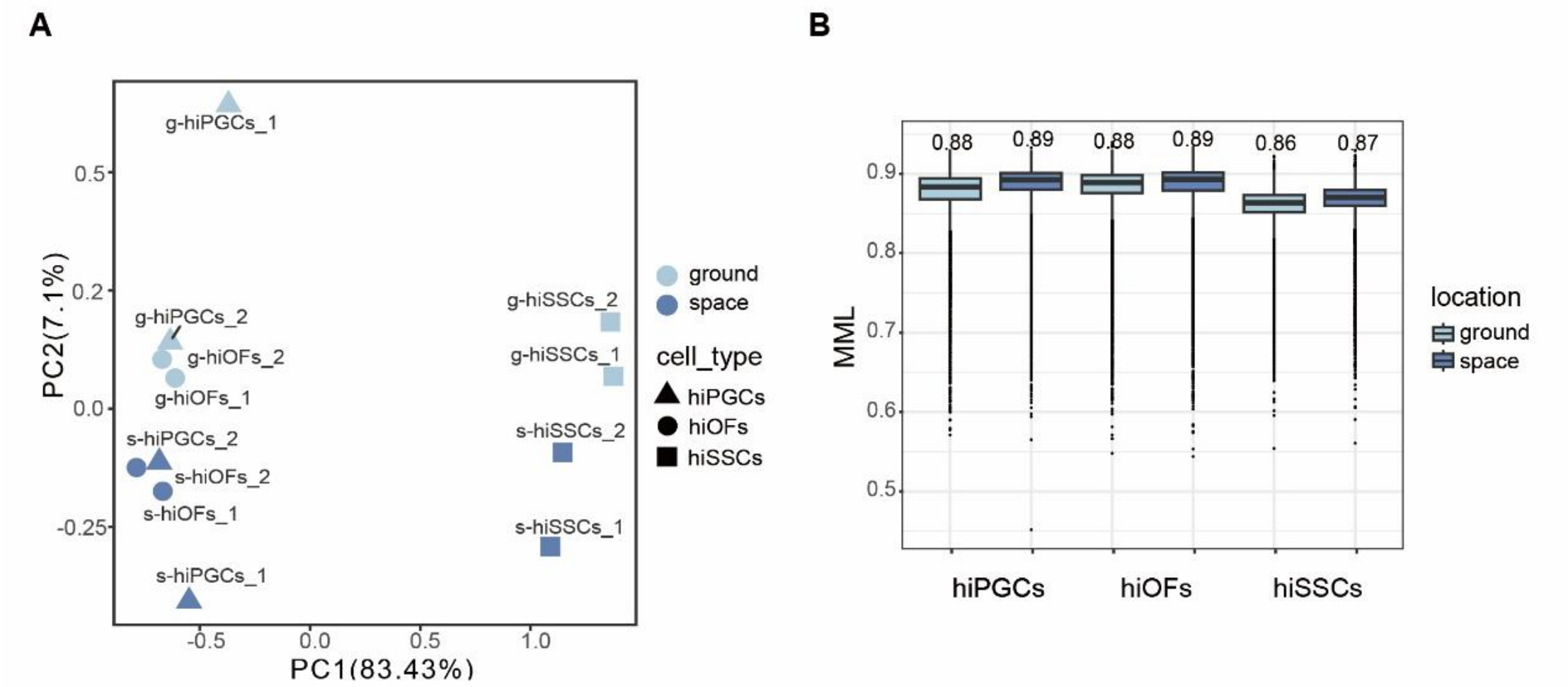

Fig. S6. Genomic and epigenomic stability under spaceflight.

(A) PCA analysis of DNA methylation profiles across three cell types under spaceflight and ground-control conditions. (B) Genome-wide DNA methylation landscapes across three cell types under spaceflight and ground-control conditions, shown as MML distributions.

Table S1. Missense mutation genes with hiPGCs, hiOFs, and hiSSCs under spaceflight and ground-control conditions detected by WES.

This table presents missense mutation genes identified by WES in hiPGCs, hiOFs, and hiSSCs under spaceflight and ground control conditions. These genes were used for the pathway enrichment analysis shown in Fig. 6J.
